# Association between statin therapy and mortality in patients on dialysis after atherosclerotic cardiovascular diseases

**DOI:** 10.1038/s41598-023-37819-1

**Published:** 2023-07-06

**Authors:** Myunhee Lee, Won Jung Choi, Yunhee Lee, Kyusup Lee, Mahn-Won Park, Jun-Pyo Myong, Dae-Won Kim

**Affiliations:** 1 Division of Cardiology, Department of Internal Medicine, Daejeon St. Mary’s Hospital, The Catholic University of Korea, Seoul, Korea; 2 Catholic Research Institute for Intractable Cardiovascular Disease CRID, College of Medicine, The Catholic University of Korea, Seoul, Korea; 3 Division of Nephrology, Department of Internal Medicine, Daejeon St. Mary’s Hospital, The Catholic University of Korea, Seoul, Korea; 4 Department of Urology, Seoul St. Mary’s Hospital, The Catholic University of Korea, Seoul, Korea; 5 Department of Occupational and Environmental Medicine, Seoul St. Mary’s Hospital, The Catholic University of Korea, Seoul, Korea

**Keywords:** Cardiology, Medical research, Nephrology

## Abstract

Statin therapy is essential for secondary prevention in patients with atherosclerotic cardiovascular disease (ASCVD). However, the effects of statin therapy in patients receiving chronic dialysis remain uncertain. We aimed to evaluate the effect of statin therapy on long-term mortality in patients on dialysis after a first-time ASCVD. Patients receiving maintenance dialysis aged ≥ 18 years with a first-time ASCVD event between 2013 and 2018 were included in the Korean National Health Insurance Service database. Associations of statin use with long-term mortality were examined using Cox proportional hazards regression models adjusted for demographics and comorbidities. Among 17,242 patients on dialysis, 9611 (55.7%) were prescribed statins after a first-time ASCVD event. Among statin users, 7376 (76.7%) used moderate-intensity statins. During a mean follow-up of 32.6 ± 20.9 months, statin use was associated with a lower risk of all-cause mortality than statin nonuse after adjusting for confounding factors (hazard ratio [HR]: 0.92; 95% confidence interval [CI] 0.88–0.97; *p* = 0.0009). Despite a lack of evidence, more than half of patients on dialysis were prescribed statins after an ASCVD event. In patients on dialysis after ASCVD, statin therapy significantly reduced the risk of long-term all-cause mortality.

## Introduction

Patients on dialysis have more atherosclerotic cardiovascular disease (ASCVD) risk factors^1^ and an unacceptably higher prevalence of ASCVD than the general population. Furthermore, cardiovascular (CV) disease is the leading cause of death in patients receiving dialysis, accounting for more than 50% of deaths in these patients^2,3^. Statin, a lipid-lowering agent, is widely used for primary and secondary prevention of ASCVD and is effective in improving CV outcomes and reducing mortality in the general population at risk of ASCVD, including patients with nondialysis-dependent chronic kidney disease (CKD)^4,5^. However, recent guidelines^6–8^ have not provided any recommendation for the initiation of statin therapy in high-risk patients on dialysis, such as those with preexisting ASCVD, because previous landmark trials have failed to prove the CV benefits^9–11^.

Despite the lack of guideline recommendations and insufficient supporting evidence of statin therapy for secondary prevention, statins have been widely prescribed in real-world practice in patients on dialysis who had high-risk profiles, such as those with diabetes or preexisting ASCVD. Additionally, current American and European guidelines recommend continuing statins in patients on dialysis who are already on statins, particularly in those with ASCVD^6,7^. Recently, some observational studies have consistently reported the mortality benefit of statin therapy in high-risk patients on dialysis, such as those with coronary heart disease or a history of coronary revascularization^12–16^. A recent retrospective study from the Japan percutaneous coronary intervention (PCI) registry demonstrated that statin therapy was associated with a reduced risk of all-cause death and cardiovascular death, irrespective of the magnitude of the low-density lipoprotein (LDL)-lowering effect^12^. Two observational studies from the Taiwan National Health Insurance Research Database showed a beneficial effect of statins in reducing the risk of all-cause mortality following myocardial infarction^15,16^. In a study of 150 Korean patients requiring chronic hemodialysis who underwent PCI, statin therapy significantly decreased the risk of the composite of nonfatal myocardial infarction, stroke, and all-cause mortality^14^.

Therefore, we hypothesized that the initiation of statin therapy after ASCVD events might significantly decrease mortality risk in patients on dialysis. This study aimed to examine our hypothesis in patients on dialysis who experienced a first-time ASCVD event using the Korean NHIS (Nationwide Health Insurance Service) database.

## Materials and methods

### Data sources and study population

This study used the Korean NHIS database. The NHIS is an obligatory health insurance system that covers over 98% of the Korean population, including approximately 50 million people^17^. The NHIS database includes all beneficiaries' medical records, including demographic data, outpatients’ visits, hospitalization, medical history, general health check-ups, procedures, prescribed medications, diagnoses, and mortality. The diagnostic code was based on the International Classification of Diseases, 10th revision (ICD-10). Among adult (age $\ge$ 18 years) patients with end-stage renal disease (ESRD) requiring maintenance dialysis, 23,317 patients who experienced a first-time ASCVD event between January 1, 2013, and December 31, 2018, were identified. The index date was defined as the date when the patient was diagnosed with ASCVD for the first time during the study period. If a patient had an admission, the index date was defined as the discharge date. To address the beneficial effect of statin therapy in patients on dialysis after a new ASCVD event, we excluded patients with preexisting ASCVD by eliminating all cases with inpatient, outpatient, or emergency room claims for ASCVD during six months preceding the cohort entry date. This study was conducted according to the Strengthening the Reporting of Observational Studies in Epidemiology (STROBE) guidelines^18^. All methods were performed in accordance with the relevant guidelines and regulations. This study was approved by the Institutional Review Board (IRB) of Daejeon St. Mary’s Hospital (IRB number: DC21ZESE0018). The IRB of Daejeon St. Mary’s Hospital waived the requirement for informed consent because the data analyses were performed retrospectively using anonymized data derived from the Korean NHIS database, which is publicly available via the official website (https://nhiss.nhis.or.kr/).

### Study design, patient selection, and definition

**Figure 1:**
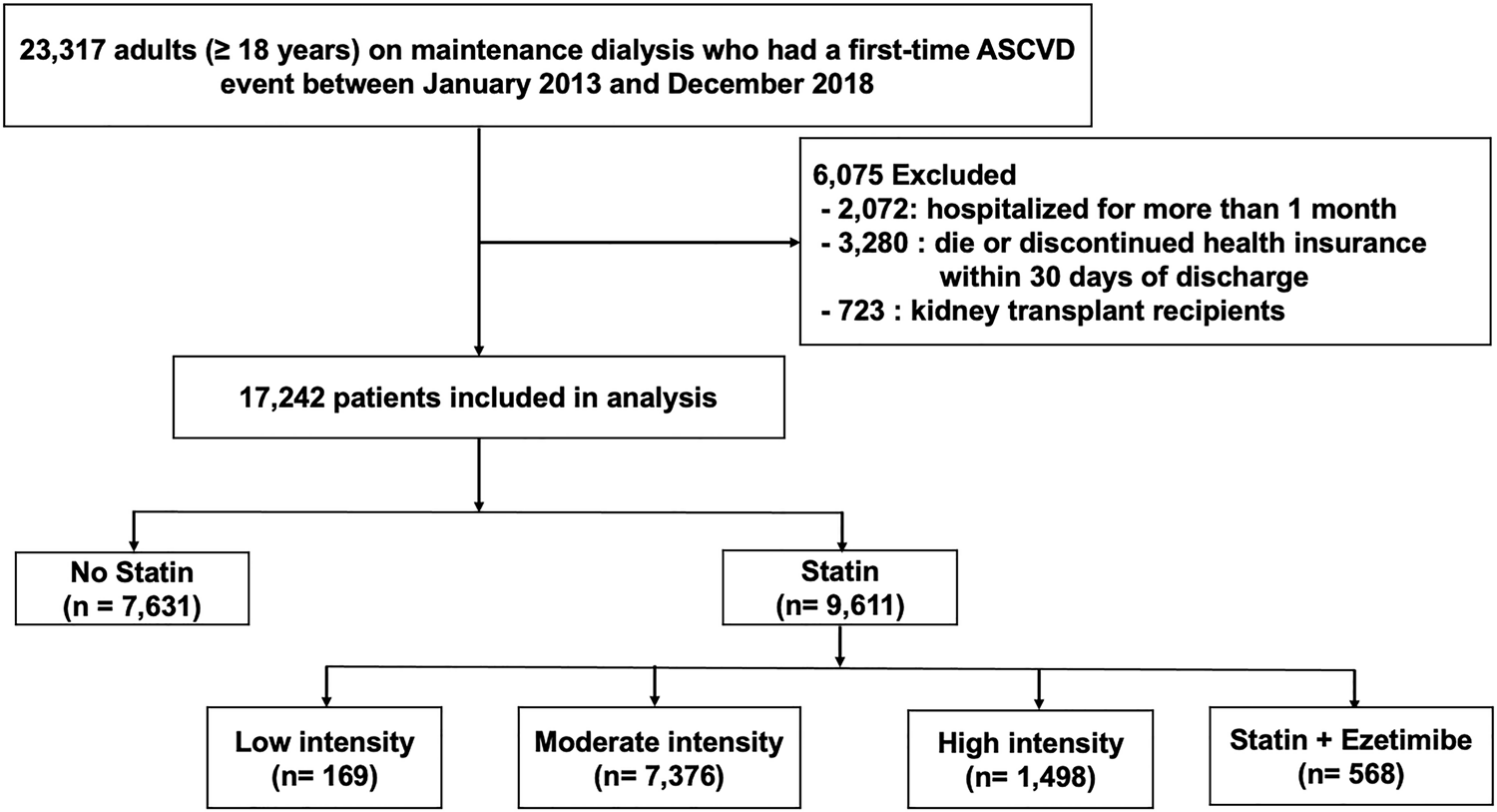
Flow chart of the study design.

Patients with ESRD undergoing maintenance dialysis were defined as those who met the following criteria: (1) had ICD-10 codes related to ESRD (N18-19) and (2) had procedure codes of hemodialysis or material codes of peritoneal dialysis fluid at least 3 months from the index date. ASCVD was defined as a composite of coronary heart disease (CHD) (myocardial infarction, angina, coronary revascularization), cerebrovascular accident (CVA) (ischemic stroke, transient ischemic attack (TIA)), or peripheral artery disease (PAD) according to the American College of Cardiology and American Heart Association (ACC/AHA) 2013 guidelines^19^. The diagnosis of ASCVD was made if the ICD-10 code for each ASCVD component was recorded with a history of admission/emergency department visit at least once or an outpatient clinic visit more than twice. Patients with angina, ischemic stroke, TIA, or PAD were required to have both ICD-10 codes for each diagnosis and additional claim codes such as imaging studies, endovascular procedures, or surgery only to include patients with clinical evidence of ASCVD. We excluded patients with ischemic stroke or TIA if they had a diagnosis of atrial fibrillation on the index date to exclude patients with a cardioembolic cause of stroke. Coronary revascularization was identified using related procedure codes. Other exclusion criteria were patients who (1) died or discontinued health insurance within 30 days of discharge, (2) had a record of a hospital stay longer than one month at index hospitalization, and (3) received renal transplantation before the index date. Finally, a total of 17,242 adult patients with ESRD who had their first ASCVD event were identified as an eligible population for the present study and were then classified into two groups according to whether they received (statin group) or did not receive (no statin group) statin therapy after the ASCVD event. Statin use was defined as a filled prescription during the first 30 days of the index date. Patients with statin use were further divided into four groups based on statin intensity (Fig. 1). The statin intensity was classified according to the ACC/AHA cholesterol treatment guideline^19^. The proportion of days covered (PDC) was used to estimate the statin adherence of the study population. We calculated the PDC by dividing the number of statin prescription days one year after the index date by 365. Patient baseline characteristics were reported on the index date. The Charlson Comorbidity Index (CCI) was calculated to assess patients' underlying medical conditions^20^. Patients were followed until December 31, 2019, or the date of an exemption from health insurance services or death, whichever occurred first. Detailed information and definitions used in this study are provided in Supplementary Table 1.

### Outcomes

The study outcomes were all-cause mortality and cardiac mortality during the follow-up period. Mortality data and cause of death were extracted from the death certificate database from Statistics Korea and matched to the medical claim data by Health Insurance and Review Assessment.

### Statistical analysis

Continuous variables were expressed as means $\pm$ standard deviation (SD) when normally distributed or as medians (interquartile range) if the hypothesis of normality was rejected. Categorical data were presented as absolute values and percentages. Differences between groups of categorical variables were analyzed by the *chi-squared or Fisher’s exact test* as appropriate. Differences between groups or continuous variables were analyzed by 2-tailed Student’s t test or the Mann–Whitney U test. The Jonckheere-Terpstra test for continuous dependent variables and Cochran Armitage test for dichotomous dependent variables were used for trend analysis. A multivariable Cox proportional hazards regression model was used to assess the association between mortality and statin use, adjusted for age, sex, index year, and CCI. The *Cox* proportional assumption was validated with a *log–log* plot and *goodness of fit* test, which satisfied this assumption. The effect of statin therapy [reference: no statin group] was expressed as hazard ratios and 95% confidence intervals. In a sensitivity analysis, we evaluated the association between statin use and mortality according to ASCVD type or statin intensity. Subgroup analyses were performed according to age group (ages < 65 years and ≥ 75 years), sex, presence of comorbidities, and CCI. The overall survival rates were compared using a Kaplan–Meier curve, and the log-rank test was used to compare the groups. A 2-sided *p* < 0.05 was considered to be statistically significant. Statistical analyses were performed using SAS software V.9.4 (SAS Institute, NC, Cary, USA).

## Results

### Baseline patient characteristics

Table 1 shows the baseline characteristics of the study population stratified by statin use and statin intensity. Baseline characteristics stratified by ASCVD type are displayed in Supplementary Table 2. A total of 9611 (55.7%) patients were prescribed statin therapy after a first-time ASCVD event. Patients prescribed statins at discharge showed good adherence, representing mean PDCs of over 80% during the first year in all four statin groups. Among statin users, 169 (1.8%) were taking low-intensity, 7376 (76.7%) were taking moderate-intensity, 1498 (15.6%) were taking high-intensity, and 568 (5.9%) were taking statin-ezetimibe combination. The mean age of the overall population was 63.0 $\pm$ 11.3 years. A total of 10,650 (61.8%) were male. Compared with statin nonusers, statin users were older and showed a higher prevalence of comorbidities such as diabetes, hyperlipidemia, hypertension, or congestive heart failure (CHF), as demonstrated by a higher mean CCI. Among 11,020 patients with CHD, 6942 (63%) were prescribed statin therapy (1.6% low-intensity, 75.3% moderate-intensity, and 17.2% high-intensity statins were prescribed, as well as 5.9% of a statin-ezetimibe combination). Patients with CVA or PAD showed similar statin prescription patterns as those with CHD; most patients received moderate-intensity statins, followed by high-intensity statins, the statin-ezetimibe combination, and low-intensity statins. Among 17,242 patients on dialysis who experienced a first-time ASCVD event, 11,020 (63.9%) patients had CHD, 2867 (16.6%) had CVA, and 3355 (19.5%) had PAD. Patients with CVA were older than those with CHD or PAD. Patients with CHD were more likely to have multiple comorbidities, such as hypertension, CHF, or chronic pulmonary disease, than patients with CVA or PAD, as demonstrated by a higher mean CCI (Supplementary Table 2).

**Table 1:** Baseline characteristics of patients on dialysis with a first time ASCVD event stratified by statin use and intensity.

	Overall (n = 17242)	No Statin (n = 7631)	Low (n = 169)	Statin moderate (n = 7376)	High (n = 1498)	+ Ezetimibe (n = 568)	*p* value
Age (year)	63.0 ± 11.3	62.8 ± 11.7	63.3 ± 11.7	63.3 ± 10.9	62.8 ± 11.2	62.2 ± 11.1	0.022
Sex (male, %)	10,650 (61.8)	4634 (60.7)	105 (62.1)	4588 (62.2)	964 (64.4)	359 (63.2)	0.005
Comorbidities (n, %)							
Diabetes with chronic complications	11,561 (67.1)	4794 (62.8)	110 (65.1)	5171 (70.1)	1077 (71.9)	409 (72.0)	< 0.0001
Diabetes	11,900 (69.0)	4896 (64.2)	118 (69.8)	5366 (72.8)	1086 (72.5)	434 (76.4)	< 0.0001
Hyperlipidemia	13,245 (76.8)	4766 (62.5)	154 (91.1)	6484 (87.9)	1318 (88.0)	523 (92.1)	< 0.0001
Hypertension	16,141 (93.6)	7075 (92.7)	155 (91.7)	6940 (94.1)	1442 (96.3)	529 (93.1)	< 0.0001
Congestive heart failure	7948 (46.1)	3177 (41.6)	72 (42.6)	3638 (49.3)	806 (53.8)	255 (44.9)	< 0.0001
Atrial fibrillation	1655 (9.6)	726 (9.5)	10 (5.9)	692 (9.4)	167 (11.2)	60 (10.6)	0.097
Chronic pulmonary disease	6614 (38.4)	2894 (37.9)	66 (39.1)	2872 (38.9)	572 (38.2)	210 (37.0)	0.705
Moderate to severe liver disease	127 (0.7)	74 (1.0)	1 (0.6)	40 (0.5)	8 (0.5)	4 (0.7)	0.035
Cancer	1853 (10.7)	856 (11.2)	21 (12.4)	758 (10.3)	165 (11.0)	53 (9.3)	0.262
PDC, %			83.2	82.4	81.5	85.3	
Follow-up duration (mean ± SD) (months)	32.6 ± 20.9	32.8 ± 21.1	36.9 ± 23.1	33.1 ± 20.9	30.0 ± 19.8	30.1 ± 19.8	
Charlson comorbidity index							
Mean	4.9 ± 1.7	4.6 ± 1.8	5.0 ± 1.7	5.2 ± 1.7	5.3 ± 1.6	5.2 ± 1.6	< 0.0001
CCI < 3	1630 (9.5)	1015 (13.3)	13 (7.7)	497 (6.7)	76 (5.1)	29 (5.1)	< 0.0001
CCI $\ge$ 3	15,612 (90.5)	6616 (86.7)	156 (92.3)	6879 (93.3)	1422 (94.9)	539 (94.9)	< 0.0001
ASCVD types (n, %)							
CHD	11,020 (63.9)	4078 (53.4)	113 (66.9)	5230 (70.9)	1191 (79.5)	408 (71.8)	< 0.0001
CVA	2867 (16.6)	1520 (19.9)	31 (18.3)	1039 (14.1)	213 (14.2)	64 (11.3)	< 0.0001
PAD	3355 (19.5)	2033 (26.6)	25 (14.8)	1107 (15.0)	94 (6.3)	96 (16.9)	< .0001

Data are mean ± SD or number (%).

PDC was defined as follows: Number of days covered by any statin prescription during 1 year after index date divided by 365.

*ASCVD* atherosclerotic cardiovascular disease, *CCI* charlson comorbidity index, *CHD* coronary heart disease, *CVA* cerebrovascular accident, *PAD* peripheral artery disease, *PDC* proportion of days covered.

### Incidence of all-cause mortality and cardiac mortality according to statin therapy

**Figure 2:**
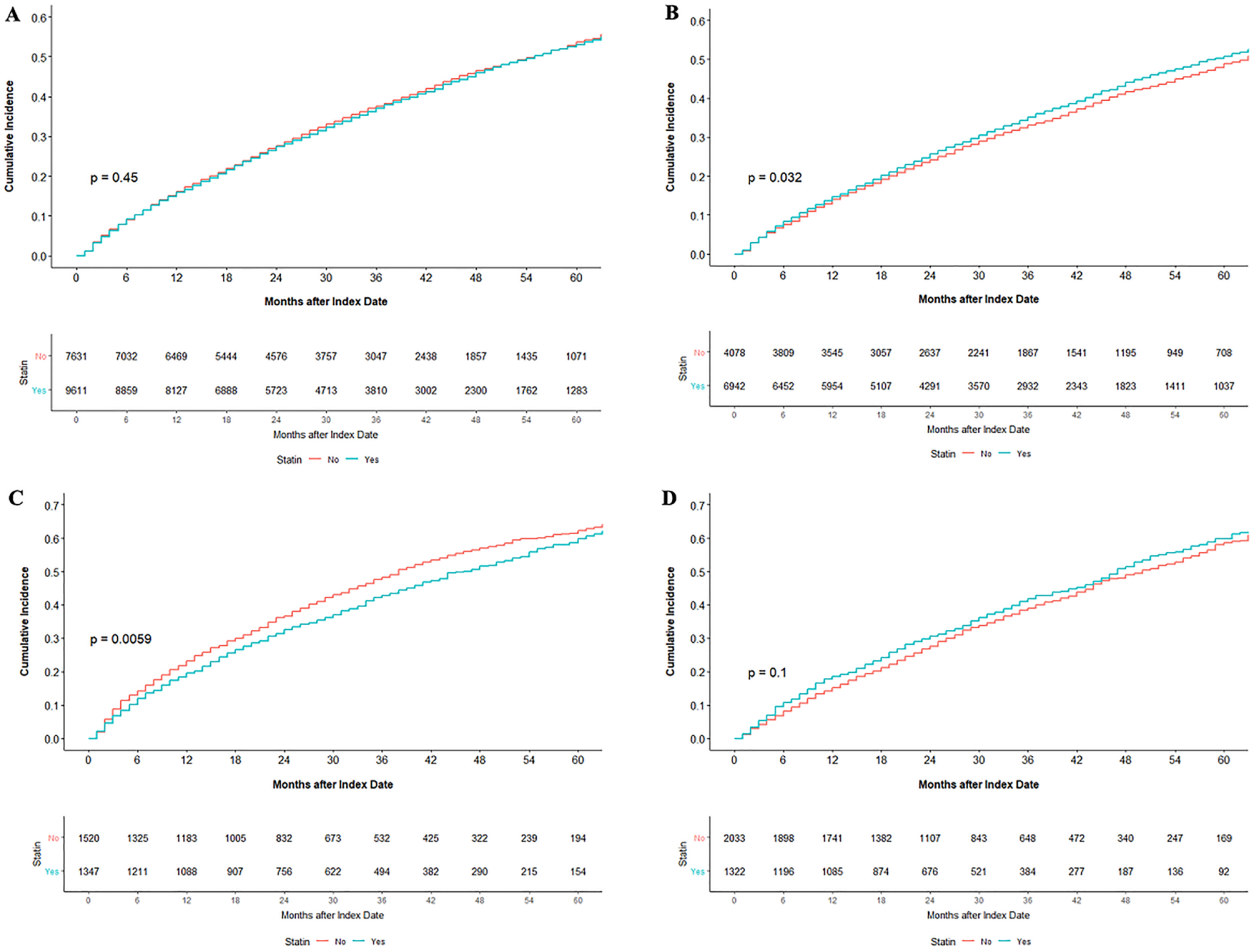
Kaplan‒Meier cumulative event curves for all-cause mortality according to statin use (**A**) in the overall population, (**B**) in patients with CHD, (**C**) CVA, or (**D**) PAD. *CHD* coronary heart disease, *CVA* cerebrovascular accident, *PAD* peripheral artery disease.

Supplementary Table 3 shows the incidence of all-cause and cardiac mortality according to statin therapy in the overall cohort and patients stratified by statin intensity. During the mean follow-up duration of 32.6 $\pm$ 20.9 months, 7275 (42.2%) patients died, and 1637 (9.5%) patients died from cardiovascular causes. The statin group showed a numerically lower incidence of all-cause mortality than the no statin group in overall and patients with CVA. However, statins were associated with a numerically increased all-cause mortality rate in patients with CHD or PAD. Cardiac mortality was higher in the statin group than in the no statin group in the overall cohort and each of the three subgroups (Supplementary Table 3). Figure 2 illustrates the cumulative incidence of all-cause mortality according to statin therapy. Overall, no difference was found in the crude mortality rate between the statin and no statin groups. In patients with CHD, statin use was associated with a higher incidence of all-cause mortality (*p* = 0.032), whereas statin use showed a marginally lower incidence rate of all-cause mortality in patients with CVA (*p* = 0.0059) (Fig. 2).

### Association of statin therapy with long-term mortality

Table 2 shows the risk of all-cause and cardiac mortality in patients on dialysis after a first-time ASCVD events during 32.6 $\pm$ 20.9 months. In the multivariable Cox analysis adjusted for age, sex, CCI, and index year, patients prescribed statins had an 8% lower risk of all-cause mortality than those who did not prescribe statins in the overall cohort (fully adjusted hazard ratio (HR): 0.92; 95% confidence interval (CI) 0.88–0.97; *p* = 0.0009). In patients with CVA, the statin group showed a 12% risk reduction of all-cause mortality compared with the no statin group (fully adjusted HR: 0.88; 95% CI 0.80–0.98; *p* = 0.021). In patients with CHD, statin therapy showed a similar trend in reducing all-cause mortality but was not statistically significant (adjusted HR: 0.97; 95% CI 0.92–1.04; *p* = 0.411). In contrast to all-cause mortality, the statin group showed a higher cardiac mortality rate than the no-statin group in both univariable and multivariable Cox regression models. However, no significant association was observed between statin therapy and cardiac mortality in dialysis patients with each ASCVD subtype.

**Table 2:** Association between statin use and mortality in patients on dialysis with a first-time ASCVD event.

	Unadjusted		Adjusted for age and sex		Fully adjusted*	
	HR (95% CI)	p value	HR (95% CI)	p value	HR (95% CI)	p value
All-cause mortality						
No statin	1 (Reference)		1 (Reference)		1 (Reference)	
Statin						
Overall	0.98 (0.94–1.03)	0.454	0.96 (0.92–1.01)	0.086	0.92 (0.88–0.97)	0.0009
CHD	1.07 (1.01–1.14)	0.035	1.01 (0.95–1.08)	0.683	0.97 (0.92–1.04)	0.411
CVA	0.87 (0.78–0.96)	0.006	0.89 (0.81–0.99)	0.034	0.88 (0.80–0.98)	0.021
PAD	1.09 (0.98–1.22)	0.107	1.10 (0.98–1.22)	0.099	1.03 (0.92–1.15)	0.598
Cardiac mortality						
No statin	1 (Reference)		1 (Reference)		1 (Reference)	
Statin						
Overall	1.16 (1.05–1.28)	0.004	1.13 (1.03–1.25)	0.014	1.12 (1.01–1.23)	0.031
CHD	1.17 (1.04–1.32)	0.012	1.12 (0.99–1.26)	0.078	1.09 (0.96–1.23)	0.170
CVA	1.00 (0.79–1.26)	0.980	1.02 (0.81–1.29)	0.847	1.05 (0.83–1.33)	0.693
PAD	1.10 (0.83–1.45)	0.502	1.10 (0.84–1.45)	0.499	1.09 (0.82–1.44)	0.565

*ASCVD* atherosclerotic cardiovascular disease, *CCI* charlson comorbidity index, *CHD* coronary heart disease, *CI* confidence interval, *CVA* cerebrovascular accident, *HR* hazard ratio, *PAD* peripheral artery disease.

*Adjusted for age, sex, CCI, index year.

### Association of statin intensities with long-term mortality

We further examined the association between statin therapy and mortality stratified by statin intensity (Table 3). Low- or moderate-intensity statin use was independently associated with reduced all-cause mortality after adjusting for age, sex, CCI, and index year in the overall population (low-intensity, fully adjusted HR: 0.76, 95% CI 0.60–0.97, *p* = 0.025; moderate-intensity, fully adjusted HR: 0.91, 95% CI 0.87–0.96, *p* = 0.0002). In patients with CVA, the moderate-intensity statin group showed a 13% (fully adjusted HR: 0.87; 95% CI 0.78–0.97; *p* = 0.013) reduction in all-cause mortality compared with the no-statin group. However, in patients with PAD, the high-intensity statin group showed a significantly higher all-cause mortality (crude HR: 1.60, 95% CI 1.21–2.10, *p* = 0.001; age- and sex-adjusted HR: 1.64, 95% CI 1.25–2.16, *p* = 0.0004; fully adjusted HR: 1.55, 95% CI 1.17–2.04, *p* = 0.002). The high-intensity statin group showed a statistically significant higher cardiac mortality than the no statin group in the overall (fully adjusted HR: 1.38; 95% CI 1.16–1.63; *p* = 0.0002) and CHD cohorts (fully adjusted HR: 1.29; 95% CI: 1.06–1.57; *p* = 0.01). Low- and moderate-intensity statin or statin/ezetimibe combination therapy showed no significant association with cardiac mortality in the univariable and multivariable Cox regression models.

**Table 3:** Association between statin intensity and mortality in patients on dialysis with a first-time ASCVD event.

(A) All-cause mortality						
	Unadjusted		Adjusted for age and sex		Fully adjusted*	
	HR (95% CI)	*p* value	HR (95% CI)	*p* value	HR (95% CI)	*p* value
Overall						
No statin	1 (Reference)		1 (Reference)		1 (Reference)	
Statin						
Low	0.86 (0.68–1.09)	0.224	0.78 (0.62–0.99)	0.04	0.76 (0.60–0.97)	0.025
Moderate	0.97 (0.93–1.02)	0.272	0.95 (0.90–0.99)	0.024	0.91 (0.87–0.96)	0.0002
High	1.08 (0.99–1.17)	0.082	1.09 (1.00–1.18)	0.062	1.04 (0.96–1.14)	0.325
+ Ezetimibe	0.91 (0.79–1.05)	0.202	0.93 (0.81–1.07)	0.311	0.89 (0.77–1.02)	0.094
CHD						
No statin	1 (Reference)		1 (Reference)		1 (Reference)	
Statin						
Low	1.00 (0.75–1.33)	0.988	0.85 (0.64–1.13)	0.27	0.84 (0.63–1.11)	0.219
Moderate	1.05 (0.99–1.12)	0.124	0.99 (0.93–1.06)	0.85	0.96 (0.90–1.02)	0.186
High	1.17 (0.06–1.30)	0.002	1.14 (1.03–1.26)	0.015	1.09 (0.98–1.21)	0.099
+ Ezetimibe	1.03 (0.87–1.22)	0.728	1.00 (0.84–1.19)	0.982	0.96 (0.80–1.13)	0.596
CVA						
No statin	1 (Reference)		1 (Reference)		1 (Reference)	
Statin						
Low	0.75 (0.44–1.28)	0.292	0.76 (0.45–1.28)	0.297	0.74 (0.43–1.25)	0.257
Moderate	0.87 (0.78–0.97)	0.011	0.88 (0.78–0.98)	0.02	0.87 (0.78–0.97)	0.013
High	0.91 (0.74–1.13)	0.394	1.03 (0.84–1.27)	0.797	1.02 (0.83–1.26)	0.858
+ Ezetimibe	0.79 (0.55–1.14)	0.21	0.90 (0.63–1.30)	0.582	0.88 (0.61–1.26)	0.477
PAD						
No statin	1 (Reference)		1 (Reference)		1 (Reference)	
Statin						
Low	0.61 (0.29–1.29)	0.198	0.61 (0.29–1.29)	0.198	0.63 (0.30–1.32)	0.220
Moderate	1.09 (0.97–1.22)	0.156	1.08 (0.96–1.21)	0.186	1.02 (0.90–1.14)	0.809
High	1.60 (1.21–2.10)	0.001	1.64 (1.25–2.16)	0.0004	1.55 (1.17–2.04)	0.002
+ Ezetimibe	0.86 (0.60–1.22)	0.397	0.92 (0.65–1.31)	0.649	0.86 (0.60–1.23)	0.404

(B) Cardiac mortality						
	Unadjusted		Adjusted for age and sex		Fully adjusted*	
	HR (95% CI)	*p* value	HR (95% CI)	*p* value	HR (95% CI)	*p* value
Overall						
No statin	1 (Reference)		1 (Reference)		1 (Reference)	
Statin						
Low	1.08 (0.68–1.73)	0.742	0.99 (0.62–1.59)	0.981	0.99 (0.62–1.57)	0.949
Moderate	1.13 (1.02–1.25)	0.023	1.10 (0.99–1.22)	0.078	1.09 (0.98–1.21)	0.127
High	1.40 (1.18–1.66)	< .0001	1.40 (1.18–1.66)	< .0001	1.38 (1.16–1.63)	0.0002
+ Ezetimibe	0.94 (0.69–1.27)	0.67	0.95 (0.70–1.29)	0.726	0.93 (0.68–1.27)	0.647
CHD						
No statin	1 (Reference)		1 (Reference)		1 (Reference)	
Statin						
Low	1.19 (0.70–2.03)	0.521	1.05 (0.61–1.78)	0.87	1.04 (0.61–1.77)	0.891
Moderate	1.15 (1.01–1.31)	0.035	1.09 (0.96–1.24)	0.175	1.07 (0.94–1.22)	0.306
High	1.38 (1.14–1.67)	0.001	1.34 (1.10–1.62)	0.003	1.29 (1.06–1.57)	0.01
+ Ezetimibe	0.86 (0.59–1.25)	0.433	0.84 (0.57–1.22)	0.348	0.81 (0.56–1.19)	0.28
CVA						
No statin	1 (Reference)		1 (Reference)		1 (Reference)	
Statin						
Low	0.59 (0.15–2.37)	0.455	0.59 (0.15–2.37)	0.453	0.58 (0.14–2.34)	0.442
Moderate	0.99 (0.77–1.27)	0.92	1.00 (0.78–1.28)	0.984	1.03 (0.80–1.32)	0.846
High	1.26 (0.83–1.93)	0.278	1.39 (0.91–2.13)	0.128	1.40 (0.91–2.15)	0.122
+ Ezetimibe	0.58 (0.22–1.57)	0.284	0.64 (0.24–1.73)	0.378	0.67 (0.25–1.81)	0.431
PAD						
No statin	1 (Reference)		1 (Reference)		1 (Reference)	
Statin						
Low	1.12 (0.28–4.55)	0.87	1.11 (0.28–4.51)	0.88	1.15 (0.28–4.66)	0.845
Moderate	1.04 (0.77–1.39)	0.819	1.03 (0.76–1.38)	0.854	1.02 (0.75–1.37)	0.922
High	1.15 (0.51–2.61)	0.74	1.19 (0.53–2.70)	0.675	1.20 (0.53–2.73)	0.669
+ Ezetimibe	1.78 (0.93–3.39)	0.08	1.89 (0.99–3.60)	0.054	1.83 (0.96–3.51)	0.069

*ASCVD* atherosclerotic cardiovascular disease, *CCI* charlson comorbidity index, *CHD* coronary heart disease, *CI* confidence interval, *CVA* cerebrovascular accident, *HR* hazard ratio, *PAD* peripheral artery disease.

*Adjusted for age, sex, CCI, index year.

### Subgroup analysis

**Figure 3:**
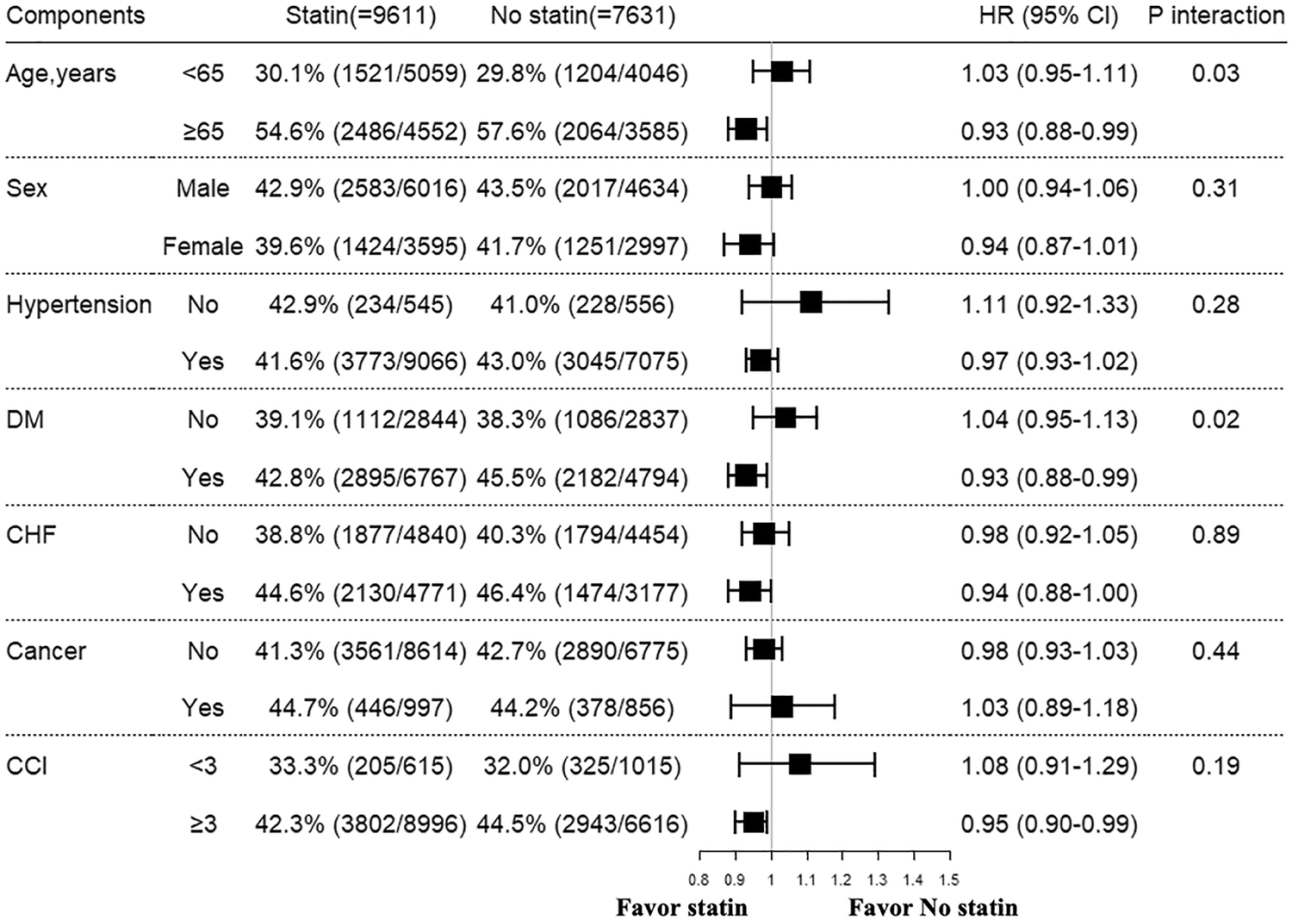
Subgroup analysis of all-cause mortality. *CHF* congestive heart failure, *DM* diabetes mellitus, *CCI* Charlson comorbidity index.

Subgroup analysis for all-cause mortality is presented in Fig. 3. Statin therapy showed a trend for reducing the risk of all-cause mortality in patients with high-risk profiles, such as older ($\ge$ 65 years old) patients, female patients, those with multiple comorbidities (such as patients with diabetes, CHF and a higher CCI value). In particular, the statin group had a significantly lower risk of all-cause mortality than the no statin group in patients with older age ($\ge$ 65 years old) and diabetes with a statistically significant interaction, implying that statin therapy could be more beneficial in these subgroups.

## Discussion

In the present study, we investigated the association of statin therapy with long-term mortality in patients on dialysis after a first-time ASCVD event. The main findings of our study can be summarized as follows. First, over half of the patients on dialysis (55.7%) were prescribed statins after a first ASCVD event, with most patients receiving moderate- or high-intensity statins (76.7% of patients taking moderate-intensity statins and 15.6% of patients taking high-intensity statins). Second, statin therapy independently reduced the risk of all-cause mortality in patients on dialysis after a first-time ASCVD event for 32.6 $\pm$ 20.9 months of follow-up. However, statin therapy showed no effect in decreasing cardiac mortality in either the overall population or each ASCVD subgroup. Third, the beneficial effect of statins on all-cause mortality is more prominent in high-risk dialysis patients, such as older patients ($\ge$ 65 years old) or those with diabetes.

Although statin therapy has proven to have substantial beneficial impacts on reducing ASCVD events and improving mortality in the general population, including patients with CKD, no guidelines exist regarding statin use in patients requiring dialysis with a higher incidence of ASCVD events and CV-related mortality than the general population because previous landmark trials have failed to demonstrate the CV benefit of statin therapy^9–11^. Based on previous landmark RCTs^9–11^, the Kidney Disease Improving Global Outcomes (KDIGO) 2013 lipid management guideline suggested neither initiating nor discontinuing statins in patients on dialysis for the primary prevention of CV disease^8^. More recently, the 2018 American College of Cardiology/American Heart Association (ACC/AHA) and 2019 European Society of Cardiology (ESC) and European Atherosclerosis Society (EAS) lipid management guidelines provided no recommendation regarding statin therapy for patients on dialysis, particularly those with ASCVD^4,7^. However, the previous three landmark RCTs could not determine the effectiveness of statin therapy in patients on dialysis after ASCVD because these RCTs included only a small percentage of patients on dialysis who had a history of ASCVD. The negative results in previous landmark RCTs were likely influenced by the inclusion of many patients who were unlikely to benefit from statin therapy. Furthermore, post hoc analyses of RCTs demonstrated that statins significantly reduced the risk of cardiac events and death in hemodialysis patients with a high risk of ASCVD, such as those with diabetes^21^, elevated LDL cholesterol^22^, or a high burden of atherosclerosis-related CV risk^23^. Currently, numerous previous studies using real-world data have also demonstrated the beneficial effects of statin use on long-term mortality in patients on dialysis who had high CV risk profiles, such as patients with a history of PCI^12,14^ or MI^13,15,16^. These findings implied that the effect of statins in patients on dialysis is heterogeneous, and a specific population with a high burden of atherosclerosis-related CV risk factors, such as patients with diabetes or a history of previous ASCVD, may benefit from statin therapy. Our study also demonstrated that statin use after a first-time ASCVD event in patients on dialysis is beneficial in reducing long-term all-cause mortality, and this beneficial effect of statins is more prominent in patients with diabetes. In contemporary clinical practice, statin prescriptions in patients on dialysis are prevalent despite the lack of guideline recommendations. Among US veterans receiving maintenance dialysis, more than half (58.1%) of patients used statins, and over one-third of statin use was high-dose^24^, a finding that is consistent with our results. This widespread use of statins in real-world practice, particularly moderate- or high-intensity statins, reflects physicians’ strong urges to prevent future ASCVD events and reduce morbidities and mortality in patients on dialysis.

One noteworthy finding of our study was that statin use was independently associated with a reduction in all-cause mortality but not cardiac mortality in patients on dialysis after a first-time ASCVD event. Previous observational studies conducted on patients requiring dialysis with a very high CV risk also showed results consistent with our study. A Taiwan nationwide cohort study demonstrated that statin therapy showed neutral effects on composite CV outcomes but significantly reduced the risk of all-cause mortality in patients with type 2 diabetes mellitus on dialysis after acute MI^15^. Another retrospective cohort study using the Korean insurance data reported that moderate- to high-intensity statins significantly lowered all-cause mortality by 24% but did not influence CV outcomes in patients on dialysis after acute MI^16^. The following hypotheses could summarize the plausible explanations for the discrepancy between all-cause and cardiac mortality on statin therapy in patients on dialysis. First, the pathogenesis of CV disease in patients on dialysis is complex and markedly different from that in the general population. Additionally, traditional risk factors for ASCVD do not have the same prognostic value in patients with CKD, particularly in patients with advanced CKD, as in the general population. Several nontraditional risk factors, such as volume overload, vascular calcification, oxidative stress, uremic toxins, inflammation, endothelial dysfunction, abnormal lipid modifications, protein-energy wasting, and abnormalities in chronic kidney disease–mineral bone disorder (CKD–MBD), are more prevalent in advanced CKD (including dialysis) and seem to play a far more critical role in the pathogenesis of ASCVD and CV mortality than in the general population^25^. The influences of these nontraditional factors should account for competing risks and might outweigh the protective effect of statins in preventing CV disease and decreasing CV mortality in patients on dialysis. Second, the beneficial cardiovascular pleiotropic effects of statins, such as increased nitric oxide bioavailability, antioxidant properties, and inhibition of inflammatory responses, work independently of LDL-cholesterol reduction and may reduce non-CV mortality in patients on dialysis^26^. Therefore, the beneficial effect of statins may not be primarily associated with reduced CV mortality in patients on dialysis.

This study has limitations. First, because of the characteristics of the observational study, potential residual biases from concomitant use of other CV protective medications or unmeasured CV risk factors, such as nutritional status, socio-economic status, accessibility to healthcare service, and smoking status, cannot be ruled out and may have influenced the results. Second, because the data used in this study are based on NHIS data that do not provide laboratory data such as lipid profiles, insufficient lipid-lowering effects with statins may affect the study results. Third, dialysis duration should be considered one of the important competing risk factors. In a study of 14,298 US veterans transitioning to dialysis, patients who recently started dialysis would most likely benefit from statins. However, the benefit diminished as the duration of dialysis became longer^27^. Unfortunately, NHIS data do not capture information about the time since the onset of dialysis for the individual patient. Fourth, the prescribed statin dose or intensity might change over time during the study period. The alternation of statin dose/intensity might influence the effect of statin on long-term mortality. Fifth, statin use before the index date was not considered in the analysis. However, we exclusively included patients who experienced a first-time ASCVD event (a clear indication for statin therapy). Therefore, the effect of statin use before the index date is not expected to be significant. Sixth, we did not evaluate cause-specific deaths, except for cardiac mortality. The potential differential misclassification effect on cardiac mortality might exist in this study. Finally, our results were based on the Korean nationwide health care database, and the limitation of diverse ethnicities can exist for our study. Despite these limitations, a strength of our study includes the large study population on a nationwide scale covering all patients on dialysis in Korea.

## Conclusions

Despite a lack of supporting evidence, statin prescription in patients on dialysis after a first-time ASCVD event is prevalent, accounting for over half of patients, and most patients received moderate- or high-intensity statins. Statin therapy might be effective in reducing long-term all-cause mortality in patients on dialysis after ASCVD. However, large scale, well-designed trials are needed to confirm our findings before they can be implemented in clinical practice.

## Supplementary Information

Supplementary Tables.

## Data Availability

The datasets generated and analyzed in this study will not be publicly available until NHIS grants permission for access to the data. The study protocol can be obtained with the permission of the corresponding author.

## Abbreviations

ASCVD: Atherosclerotic cardiovascular disease
CHD: Coronary heart disease
CKD: Chronic kidney disease
CVA: Cerebrovascular accident
ESRD: End-stage renal disease
NHIS: Nationwide health insurance service
PAD: Peripheral artery disease
