# Supplementary material for: Association between statin therapy and mortality in patients on dialysis after atherosclerotic cardiovascular diseases
Source: Sci Rep. 2023 Jul 6;13:10940. doi: 10.1038/s41598-023-37819-1 (PMC10326072; doi:10.1038/s41598-023-37819-1)
Supplement: Supplementary file 1 — Supplementary Tables. [file 41598_2023_37819_MOESM1_ESM.docx]

**Association between statin therapy and mortality in patients on dialysis after atherosclerotic cardiovascular diseases**

Myunhee Lee ^1, 2^, Won Jung Choi ^3^, Yunhee Lee ^4^, Kyusup Lee ^1, 2^, Mahn-Won Park ^1, 2^, Jun-Pyo Myong ^5, *^, and Dae-Won Kim ^1, 2, *^

^1^ Division of Cardiology, Department of Internal Medicine, Daejeon St. Mary's Hospital, The Catholic University of Korea, Seoul, Korea

^2^ Catholic Research Institute for Intractable Cardiovascular Disease CRID, College of Medicine, The Catholic University of Korea, Seoul, Korea

^3^ Division of Nephrology, Department of Internal Medicine, Daejeon St. Mary's Hospital, The Catholic University of Korea, Seoul, Korea

^4^ Department of Urology, Seoul St. Mary's Hospital, The Catholic University of Korea, Seoul, Korea

^5^ Department of Occupational and Environmental Medicine, Seoul St. Mary's Hospital, The Catholic University of Korea, Seoul, Korea

This appendix has been prepared by the authors to provide readers with additional information about the manuscript.

**^*^Corresponding Author:**

Dae-Won Kim, MD PhD

Assistant Professor, Division of Cardiology, Department of Internal Medicine, Daejeon St. Mary’s Hospital, The Catholic University of Korea, Seoul, Republic of Korea

64, Daeheung-ro, Jung-gu, Daejeon, Korea, 34943

[mirinesilver@catholic.ac.kr](mailto:mirinesilver@catholic.ac.kr)

Jun-Pyo Myong, MD PhD

Professor, Department of Occupational and Environmental Medicine, Seoul St. Mary's Hospital, The Catholic University of Korea, Seoul, Korea

222 Banpo-daero, Seocho-gu, Seoul, Korea, 06591

[jpmyong@gmail.com](mailto:jpmyong@gmail.com)

Supplementary Table legends

**Supplementary Table 1.** Definitions of variables and outcomes

**Supplementary Table 2.** Baseline characteristics according to the ASCVD subtypes in patients on dialysis

**Supplementary Table 3.** Incidence of outcomes stratified by statin use and ASCVD type in patients on dialysis

**Supplementary Table 1.** Definitions of variables and outcomes

| **Variables** | **ICD-10 Code** | **Procedure/Diagnosis Code** | **Definition** |
| --- | --- | --- | --- |
| Chronic kidney disease / ESRD | N18, N181–N185, N189, N19, Z490, Z491, Z90, or Z905 |  |  |
| Hemodialysis |  | O7011–O7018, O7020, O7021 |  |
| Peritoneal dialysis |  | 321400BIS,321600BIS,321800BIS,324000BIS,324800BIS,325900BIS,326000BIS,349700BIS,349800BIS,349900BIS,350000BIS,350200BIS,350300BIS,352100BIS,352400BIS,352800BIS,365900BIS,366000BIS,366100BIS,366200BIS,366300BIS,423200BIS,423400BIS,423500BIS,431100BIS,431200BIS,431300BIS,449000BIS,449100BIS,449200BIS,449800BIS,449900BIS,450000BIS,463700BIS,463900BIS,464000BIS,464700BIS,464800BIS,494900BIS,495000BIS,495100BIS,509600BIS,509700BIS,509800BIS,510700BIS,510800BIS,511000BIS,513500BIS,644300BIJ,665700BIS,665800BIS,665900BIS |  |
| Angina | I20.x | HA524, HC292, HC297, HC298, HC301-5, EB434, EB435, E654, HC080-HC089, HA670 | (ICD-10 code) AND (Procedure or diagnosis code) AND (Admission or ER visit > 1 or outpatient clinic $\geq$ 2) |
| Myocardial infarction | I21.x – I22.x |  | (ICD-10 code) AND (Admission or ER visit > 1) |
| Coronary revascularization | I20-I25 | M6551, M6552, M6561-4, M6571, M6572, O1641, O1642, O1647, OA641, OA642, OA647 | (ICD-10 code) AND (Procedure code) AND (Admission or ER visit > 1) |
| Ischemic stroke | I63 | HA851, HA441, HA451, HA461, HE135, HE235, HE535, HA471, HE101, HE201, HE301, HE401, HE501 | (ICD-10 code) AND (Diagnosis code) AND (Admission or ER visit > 1) |
| Transient ischemic attack | G458, G459 | HA851, HA441, HA451, HA461, HE135, HE235, HE535, HA471, HE101, HE201, HE301, HE401, HE501 | (ICD-10 code) AND (Diagnosis code) AND (Admission or ER visit > 1), exclude if ICD-code for AF (I48.x) exist at index date |
| Peripheral artery disease | I70.2, I70.3, I70.9, I73.1, I73.8, I73.9 | M6597, M6605, M6613, M6632, M6620, O1643-4, O0161~O0171, O1645~6, N0572, N0573, N0574, N0575 | (ICD-10 code) AND (Diagnosis or Procedure code) AND (Admission or ER visit > 1 or outpatient clinic $\geq$ 2) |
| Cardiac mortality | I00 – I99 |  | Recorded as a major cause of death |

| Diagnosis for Charlson Comorbidity Index | Weight | ICD-10 Code |
| --- | --- | --- |
| Congestive Heart Failure | 1 | I43, I50, I099, I110, I130, I132, I255, I420, I425, I426, I427, I428, I429, P290 |
| Chronic Pulmonary Disease | 1 | J40, J41, J42, J43, J44, J45, J46, J47, J60, J61, J62, J63, J64, J65, J66, J67, I278, I279, J684, J701, J703 |
| Diabetes without complications | 1 | E100, E101, E106, E108, E109, E110, E111, E116, E118, E119, E120, E121, E126, E128, E129, E130, E131, E136, E138, E139, E140, E141, E146, E148, E149 |
| Diabetes with complications | 2 | E102, E103, E104, E105, E107, E112, E113, E114, E115, E117, E122, E123, E124, E125, E127, E132, E133, E134, E135, E137, E142, E143, E144, E145, E147 |
| Cancer | 2 | C00, C01, C02, C03, C04, C05, C06, C07, C08, C09, C10, C11, C12, C13, C14, C15, C16, C17, C18, C19, C20, C21, C22, C23, C24, C25, C26, C30, C31, C32, C33, C34, C37, C38, C39, C40, C41, C43, C45, C46, C47, C48, C49, C50, C51, C52, C53, C54, C55, C56, C57, C58, C60, C61, C62, C63, C64, C65, C66, C67, C68, C69, C70, C71, C72, C73, C74, C75, C76, C81, C82, C83, C84, C85, C88, C90, C91, C92, C93, C94, C95, C96, C97 |
| Moderate or Severe Liver Disease | 3 | K704, K711, K721, K729, K765, K766, K767, I850, I859, I864, I982 |
| Hypertension | 1 | I10 |
| Dyslipidemia | 1 | E78 |
| Atrial fibrillation | 1 | I48 |

**Supplementary Table 2.** Baseline characteristics according to the type of ASCVD in patients on dialysis

|  | **Overall (n=17,242)** | **CHD**  **(n=11,020)** | **CVA**  **(n=2,867)** | **PAD**  **(n=3,355)** | **P-value** |
| --- | --- | --- | --- | --- | --- |
| **Index year** |  |  |  |  |  |
| 2013 | 2,633 (15.3) | 1,780 (67.6) | 507 (19.3) | 346 (13.1) | <.001 |
| 2014 | 2,636 (15.3) | 1,811 (68.7) | 441 (16.7) | 384 (14.6) | <.001 |
| 2015 | 2,594 (15.0) | 1,730 (66.7) | 445 (17.2) | 419 (16.2) | <.001 |
| 2016 | 3,078 (17.9) | 1,969 (64.0) | 497 (16.2) | 612 (19.9) | <.001 |
| 2017 | 3,194 (18.5) | 1,909 (59.7) | 537 (16.8) | 748 (23.4) | <.001 |
| 2018 | 3,107 (18.0) | 1,821 (58.6) | 440 (14.2) | 846 (27.2) | <.001 |
| Age | 63.0 ± 11.3 | 62.7 ± 11.1 | 64.8 ± 11.1 | 62.5 ± 11.9 | <.001 |
| Male sex | 10,650 (61.8) | 6,882 (62.5) | 1,718 (59.9) | 2,050 (61.1) | 0.019 |
| **Comorbidities (n, %)** |  |  |  |  |  |
| Diabetes | 11,900 (69.0) | 7,480 (67.9) | 2,002 (69.8) | 2,418 (72.1) | <.001 |
| Diabetes with chronic complications | 11,561 (67.1) | 7,439 (67.5) | 1,946 (67.9) | 2,176 (64.9) | 0.010 |
| Hyperlipidemia | 13,245 (76.8) | 8,969 (81.4) | 2,024 (70.6) | 2,252 (67.1) | <.001 |
| Hypertension | 16,141 (93.6) | 10,450 (94.8) | 2,686 (93.7) | 3,005 (89.6) | <.001 |
| Congestive heart failure | 7,948 (46.1) | 5,957 (54.1) | 920 (32.1) | 1,071 (31.9) | <.001 |
| Atrial fibrillation | 1,655 (9.6) | 1,320 (12.0) | 0 | 335 (10.0) | <.001 |
| Chronic pulmonary disease | 6,614 (38.4) | 4,384 (39.8) | 1,023 (35.7) | 1,207 (36.0) | <.001 |
| Moderate to severe liver disease | 127 (0.7) | 79 (0.7) | 27 (1.0) | 21 (0.6) | 0.321 |
| Cancer | 1,853 (10.8) | 1,210 (11.0) | 284 (9.9) | 359 (10.7) | 0.253 |
| **Charlson comorbidity index** |  |  |  |  |  |
| mean, SD | 4.93 ±1.74 | 5.09 ±1.73 | 4.62 ±1.67 | 4.67 ±1.78 | <.001 |
| category, n (%) |  |  |  |  |  |
| CCI < 3 | 1,630 (9.5) | 866 (7.9) | 350 (12.2) | 414 (12.3) | <.001 |
| CCI ≥ 3 | 15,612 (90.5) | 10,154 (92.1) | 2,517 (87.8) | 2,941 (87.7) | <.001 |

Data are mean ± SD or number (%).

ASCVD atherosclerotic cardiovascular disease, CCI charlson comorbidity index, CHD coronary heart disease, CVA cerebrovascular accident, PAD peripheral artery disease, SD standard deviation.

**Supplementary Table 3.** Incidence of outcomes stratified by statin use and ASCVD type in patients on dialysis

(Follow up duration (months) (mean $\pm$ SD): $32.6\pm20.9$ )

| **Overall** | **Overall**  **(n=17,242)** | **No Statin**  **(n=7,631)** | **Statin**  **(n=9,611)** | **Low**  **(n= 169)** | **Statin**  **Moderate**  **(n= 7,376)** | **High (n=1,498)** | **+Ezetimibe (n=568)** |
| --- | --- | --- | --- | --- | --- | --- | --- |
|  |  |  |  |  |  |  |  |
| All-cause mortality | 7275(42.2) | 3268(42.8) | 4007(41.7) | 70(41.4) | 3097(42.0) | 635(42.4) | 205(36.1) |
| Cardiac mortality | 1637(9.5) | 670(8.8) | 967(10.1) | 18(10.7) | 737(10.0) | 169(11.3) | 43(7.6) |
|  |  |  |  |  |  |  |  |

**(a) CHD**

| **CHD** | **Overall**  **(n=11,020)** | **No Statin**  **(n=4,078)** | **Statin**  **(n=6,942)** | **Low**  **(n= 113)** | **Statin**  **Moderate**  **(n= 5,230)** | **High (n=1,191)** | **+Ezetimibe (n=408)** |
| --- | --- | --- | --- | --- | --- | --- | --- |
|  |  |  |  |  |  |  |  |
| All-cause mortality | 4444 (40.3) | 1631 (40.0) | 2813(40.5) | 49 (43.4) | 2140 (40.9) | 481 (40.4) | 143 (35.0) |
| Cardiac mortality | 1143 (10.4) | 395 (9.7) | 748(10.8) | 14 (12.4) | 567 (10.8) | 138 (11.6) | 29 (7.1) |

**(b) CVA**

| **CVA** | **Overall**  **(n=2,867)** | **No Statin**  **(n=1,520)** | **Statin**  **(n=1,347)** | **Low**  **(n= 31)** | **Statin**  **Moderate**  **(n= 1,039)** | **High (n=213)** | **+Ezetimibe (n=64)** |
| --- | --- | --- | --- | --- | --- | --- | --- |
|  |  |  |  |  |  |  |  |
| All-cause mortality | 1462(51.0) | 820(53.9) | 642(47.7) | 14(45.2) | 498(47.9) | 100(46.9) | 30(46.9) |
| Cardiac mortality | 283(9.9) | 149(9.8) | 134(9.9) | 2(6.5) | 103(9.9) | 25(11.7) | 4(6.3) |

**(c) PAD**

| **PAD** | **Overall**  **(n=3,355)** | **No Statin**  **(n=2,033)** | **Statin**  **(n=1,322)** | **Low**  **(n= 25)** | **Statin**  **Moderate**  **(n= 1,107)** | **High (n=94)** | **+Ezetimibe (n=96)** |
| --- | --- | --- | --- | --- | --- | --- | --- |
|  |  |  |  |  |  |  |  |
| All-cause mortality | 1369(40.8) | 817(40.2) | 552(41.8) | 7(28.0) | 459(41.5) | 54(57.4) | 32(33.3) |
| Cardiac mortality | 211(6.3) | 126(6.2) | 85(6.4) | 2(8.0) | 67(6.1) | 6(6.4) | 10(10.4) |

Data are number (%).

ASCVD atherosclerotic cardiovascular disease, CHD coronary heart disease, CVA cerebrovascular accident, PAD peripheral artery disease.
